# The Effectiveness of Low-dose Dexmedetomidine Infusion in Sedative Flexible Bronchoscopy: A Retrospective Analysis

**DOI:** 10.3390/medicina56040193

**Published:** 2020-04-23

**Authors:** Sheng Hua Wu, David Vi Lu, Chun Dan Hsu, I Cheng Lu

**Affiliations:** 1 Department of Anesthesiology, College of Medicine, Kaohsiung Medical University, Kaohsiung 807, Taiwan; elsawu2@gmail.com; 2 Department of Anesthesiology, Kaohsiung Medical University Hospital, Kaohsiung 807, Taiwan; 3 Department of Anesthesiology, Kaohsiung Municipal Ta-Tung Hospital, Kaohsiung 807, Taiwan; 4 Department of Anesthesiology, Kaohsiung Municipal Siaogang Hospital, Kaohsiung 807, Taiwan; david1628@gmail.com (D.V.L.); mstg1103@gmail.com (C.D.H.)

**Keywords:** flexible bronchoscopy, procedural sedation, dexmedetomidine, midazolam

## Abstract

*Background and objectives:* Flexible bronchoscopy has been widely used for diagnosis and intervention, while various drugs are used for sedation during bronchoscopy. We examined two regular standardized sedation options (with or without dexmedetomidine) regularly used in our regional hospital. The aim was to assess the efficacy and safety of dexmedetomidine on conscious sedation under bronchoscopy. *Materials and Methods:* A retrospective chart review was conducted from April 2017 to March 2018. All patients undergoing flexible bronchoscopy with moderate sedation were enrolled. Patients having received dexmedetomidine-propofol-fentanyl were defined as group D, and those having received midazolam-propofol-fentanyl were defined as group M. The primary outcome was a safety profile during the procedure, including the incidence of procedural interference by patient cough or movement, transient hypoxemia, and hypotension. The secondary outcome was measured by the recovery profile (awake and ambulation time). *Results:* Thirty-five patients in group D and thirty-three in group M were collected in this retrospective study. All patients underwent the procedure successfully. Group D showed higher safety with fewer procedural interference incidences by cough or body movement than Group M (3.3% versus 36.3%, *p* < 0.001) and minor respiratory adverse effects. Patients in group D showed faster recovery in a shorter ambulation time than group M (24.9 ± 9.7 versus 31.5 ± 11.9, *p* = 0.02). In group D, bronchoscopist satisfaction to sedation was higher than group M (*p* = 0.01). More transient bradycardia episodes were noted in patients receiving dexmedetomidine (*p* < 0.05), but all recovered without atropine intervention. Overall post-procedural adverse events and satisfaction were comparable in the two groups. *Conclusions:* The co-administration of dexmedetomidine met the safety and recovery demands of flexible bronchoscopy. Compared to the conventional midazolam-propofol-fentanyl regimen, the application of dexmedetomidine improved sedative effectiveness with less procedural interruptions, shorter time to ambulation and higher bronchoscopist satisfaction.

## 1. Introduction

Flexible bronchoscopy has been widely used to assist diagnosis and intervention of pulmonary disorders. Previously, many bronchoscopists have preferred performing the procedure under topical anesthesia to avoid respiratory depression or unstable hemodynamic status during sedation. However, bronchoscopic examination under topical anesthesia might lead to patient discomfort and interrupt the performance of the bronchoscopy, especially during complex procedures.

Minor complications have been found during the bronchoscopy under sedation, such as hypoxemia [1]. Increasing evidence suggests using pharmacological sedatives to achieve moderate sedation during the procedure could achieve better patient satisfaction and improve procedural tolerance unless contraindications exist [2]. Various sedation regimens have been used to provide conscious sedation during bronchoscopy, including midazolam, short-acting opioids and propofol [3,4,5,6].

Midazolam is the most commonly used sedative for procedural sedation with minimal hemodynamic effect. However, it might depress the respiratory drive even under small standard doses, especially in the elderly [7]. Dexmedetomidine is a selective alpha 2-agonist and is emerging as a promising alternative sedative agent in procedural sedation [8]. It exhibits hypnotic and analgesic properties with minimal respiratory effects [9]. One systemic review study suggested dexmedetomidine exerts potential benefits over midazolam in procedural sedation [10]. However, these sedatives alone often provide insufficient sedative or analgesic effects as well as delayed recovery [11,12,13], for which previous studies suggest a combined sedative/analgesic regimen improve efficacy and safety during endoscopic examination [14,15,16].

Till now, there is no standardized pharmacological sedative suggested for bronchoscopy. Two sedative options are commonly used in our regional hospital for conscious sedation of bronchoscopy, including dexmedetomidine-propofol-fentanyl and midazolam-propofol-fentanyl. The purpose of our retrospective study was to analyze the efficacy and safety of a dexmedetomidine-based sedative regimen in comparison with the conventional midazolam-based regimen during flexible bronchoscopy.

## 2. Materials and Methods

This study was approved by the Institutional Review Board of Kaohsiung Medical University Hospital (Kaohsiung, Taiwan, protocol number KMUHIRB-E(I)-20190221), and written informed consent was waived for retrospective design. The protocol was also registered on ClinicalTrials.gov (NCT04169685). The analysis reviewed adult patients who underwent elective flexible bronchoscopy with moderate sedation between April 2017 and March 2018.

We included elective bronchoscopic procedures performed under total intravenous anesthesia without tracheal intubation and immediate postprocedural ambulation. Patients under the age of 18, with physical status defined as an American Society of Anesthesiologist (ASA) physical status of 4, and cases transferred to the intensive care unit, were excluded from the study. We reviewed anesthesia records of consecutive bronchoscopy and further divided the cases into two groups based on sedatives, either dexmedetomidine-(group D) or midazolam-based (group M).

A nasal cannula delivered 2 L/min of oxygen to the patient on arrival at the examination room. Routine physical monitoring was applied during sedation, including electrocardiography (lead II), heart rate, peripheral oxygen saturation, capnography, and noninvasive blood pressure (every 5 min). Abdominal wall and chest excursions were observed as an alternative method to monitor ventilation.

Baseline vital signs were recorded for all patients before sedation. A peripheral intravenous catheter was inserted in the forearm for the administration of intravenous sedatives and analgesics.

Two experienced bronchoscopists assisted by a technician performed the flexible bronchoscopy. An experienced anesthesiologist and nurse anesthetist were responsible for sedation, physical monitoring and interventions when necessary, such as for adjusting the oxygen flow to provide adequate oxygenation, jaw lifting to maintain patent airways, mask ventilation for persistent desaturation or fluid resuscitation and vasopressor for hypotension. All bronchoscopists and anesthesia staffs were qualified for advanced cardiac life support. An external defibrillator and resuscitation drugs were also available.

Dexmedetomidine-based sedative protocol included a loading dose of 0.7 μg/kg dexmedetomidine for 10 min, followed by a maintenance dose of 0.07 μg/kg/h. Midazolam-based sedative protocol included midazolam 1 mg initially, and repeated 1 mg midazolam bolus was administered to achieve adequate sedation by clinical judgement. After that, all patients received a bolus of fentanyl 25–50 μg followed by propofol target-controlled infusion (TCI) by the Orchestra Base Primea (Fresenius, Brezins, France) during the procedure. The initial effect site concentration (Ce) of propofol TCI was 3.0 mg/mL before bronchoscopy passing the vocal cord. Once bronchoscopy advanced into the trachea, the bronchoscopist administered a topical spray of 2% lidocaine 2–3 mL. The propofol infusion was titrated based on clinical judgment to achieve moderate sedation (responds only after mild prodding or mild shaking). Cough was routinely prevented by secretion suction and 2 mL 2% lidocaine administered via the bronchoscope. If persistent cough or body movement interrupted the procedure, a bolus of 25–50 μg fentanyl was administered.

All data were obtained from anesthesia documents within the chart and analyzed by an investigator blinded to our study. Patient characteristics included demographic data, ASA physical status, disease diagnosis, bronchoscopic procedure, anesthesia and procedure time. Anesthetic records included sedative regimen (each drug dosage), cardio-respiratory adverse events (observed apnea, hypoxemia, hypotension, bradycardia) and airway management, such as jaw lifting, mask ventilation, or tracheal intubation. Bronchoscopic procedures included examination only or with biopsy by forceps. Bronchoalveolar lavage (BAL) was routinely done for all at the end of examination. After the procedure was complete, the operator answered a satisfaction questionnaire on a 4-point scale (1: poor, 2: fair, 3: good, 4: excellent). After orientation was intact, the patients answered a questionnaire of post-procedural events, such as nausea, vomiting, dizziness, recall and pain. Patient satisfaction to the entire procedure on a 4-point scale (1: poor, 2: fair, 3: good, 4: excellent) was recorded by an investigator blinded to groupings.

The primary outcome was measured by the safety profile, while the secondary outcomes were measured by the recovery profile. A safety profile was evaluated by tolerability during the procedure, observed apnea, transient hypoxemia and hypotension. Tolerability was measured as cough or body movement to interrupt the procedure. We calculated the incidence of procedure interruption that the bronchoscopist had to temporarily pause the procedure to wait for intervention by physical restraint or titrating medication. Hypoxemia was defined as any episode of SpO2 less than 90% during the procedure. Hypotension was defined as systolic blood pressure less than 90 mmHg. Bradycardia was defined as heart rate less than 60 beats per minute. The recovery profile was assessed by time awake from anesthesia and ambulation time. Awake time was defined as the time consumption from the end of bronchoscopy to spontaneous eye opening and recall identity. Ambulation time was defined as time consumption from awake to walking without assistance.

All data were presented as mean ± standard deviation. A power analysis identified a minimal sample size of 27 patients per group to demonstrate a 30% difference in procedure stop with a power of 0.8 and a type-1 error of 0.05. Therefore, we reviewed over 30 valid records in each group to allow for study error and attrition.

Statistical analysis was carried out using two-sample t-tests (numerical variables) and Chi-square tests (categorical variables). The SPSS version 14.0 statistical software package (SPSS Inc., Chicago, IL, USA) was used for all analyses, and *p* values < 0.05 were considered statistically significant.

## 3. Results

We reviewed 80 consecutive patients undergoing sedative flexible bronchoscopy over a 12-month duration. Three cases failed to meet the criteria (two transferred to the intensive care unit and one discontinued due to persistent hypoxemia), and nine cases were excluded owing to incomplete data. Documentation of 35 and 33 patients in the groups D and group M were successfully analyzed, respectively. Patient characteristics of both groups are shown in Table 1. There was no significant difference in physical data, diagnosis, bronchoscopic procedures, examination time, baseline oxygenation, heart rate and blood pressure. Nearly 70% of the patients were ASA class 3 and the mean duration of the bronchoscopic procedure was 20 min. Sedation time was significantly longer in group D than in group M (*p* < 0.05) (Table 1).

Group D showed higher tolerability with less procedure interruptions by cough or body movement than group M (3.3% versus 36.3%, *p* < 0.001) (Table 2); however, the incidence of bradycardia was higher in group D than in group M (20% versus 6.1%, *p* = 0.03) (Table 2). All of the bradycardia cases recovered spontaneously after titration of dexmedetomidine or propofol without atropine administration. The incidence of transient hypoxemia, hypotension and airway management were comparable in both groups. In group D, bronchoscopist satisfaction to sedation was higher than in group M (*p* = 0.01). Patients in group D showed faster recovery in a shorter time to ambulation than in group M (24.9 ± 9.7 versus 31.5 ± 11.9, *p* = 0.02). Peri-procedural adverse events and patient satisfaction did not differ significantly between groups (Table 3).

## 4. Discussion

In our retrospective study, combination with dexmedetomidine might increase patient tolerability during the bronchial procedure and improve bronchoscopist satisfaction. In addition, it also accelerated patient recovery to ambulation without adverse effects of respiratory depression. Although more cases showed bradycardia episodes in group D than in group M, all bradycardia episodes recovered spontaneously without medication. We suggest that a combined sedation regimen of dexmedetomidine-fentanyl-propofol is feasible to facilitate the bronchoscopic procedure with less interventions to control cough/movement and shorter recovery time to ambulation when compared to the midazolam-fentanyl-propofol regimen.

Various sedatives either alone or in combination usage have been reported in bronchoscopic procedures [6,17]. Benzodiazepines exert an amnestic effect and are a commonly used agent. Opioids offer the antitussive and analgesic properties; while propofol is also effective and has become popular in procedural sedation for its fast onset and recovery profile. Co-administration of these drugs is suggested to improve patient tolerance and achieve sedation with a smaller required total dose, when feasible. A retrospective analysis showed that a triple sedation regimen (midazolam + fentanyl + propofol) provided safety and reduced dosage of sedatives [6]. Our previous study in sedative gastrointestinal endoscopy also revealed less recovery time in patients who received the triple regimen (midazolam+ fentanyl+ propofol) [12].

Dexmedetomidine is emerging as a new alternative sedation for nonintubated procedures. It has dose-dependent sedation without respiratory depression and is increasingly popular for analgesic potential by reducing the sympathetic tone [18,19]. Small-dose dexmedetomidine infusion provided sedation and analgesia as well as preserved cardiorespiratory function in healthy volunteers [20]. For its high affinity to α2-adrenoceptor, low-dose infusion of dexmedetomidine may attenuate surgical stressors and offer benefits in patients at risk for cardiac morbidities [9,21]. According to one systemic literature review study, dexmedetomidine has several advantages over midazolam when used for procedural sedation, including higher patient and operator satisfaction as well as similar respiratory and hemodynamic profiles [10]. The authors suggest dexmedetomidine is a potential alternative to midazolam for procedural sedation, if carefully titrated.

In our study, fewer interventions were needed in group D to control cough or movement during the procedure, and high satisfaction of bronchoscopists was also noted in group D. Furthermore, oxygen desaturation is a common complication undergoing bronchoscopy. A randomized double-blind study reported few incidences of desaturation in dexmedetomidine-propofol compared to remifentanil-propofol sedatives during bronchoscopy [22]. Our results showed no significant difference in hypoxemic episodes between both groups. Some reports suggest the use of dexmedetomidine may be associated with cardiovascular depression (such as bradycardia and hypotension), even leading to pulseless electrical activity [23,24]. Although an episode of bradycardia was identified more frequently in group D than in Group M, it was transient and clinically insignificant. When heart rate fell below 60 beats/min, we reduced the dose of dexmedetomidine to avoid further exacerbating the bradycardia. The incidence of hypotension was also not significantly different in both groups. Some studies mentioned that the use of dexmedetomidine is associated with longer recovery time; however, the time awake from anesthesia in a dexmedetomidine-fentanyl-propofol sedative regimen was not significantly prolonged. The reason might be associated with a smaller infusion dosage of dexmedetomidine (0.7 mcg/kg) than the standard recommendation (1 mcg/kg). Since then, we found that significant bradycardia occurred frequently in the Taiwanese population.

The study has some limitations that should be considered. Firstly, the design was not a randomized-controlled prospective study. In group D, the dexmedetomidine protocol was fixed and propofol was titrated during sedation. Secondly, limited cases were collected in our hospital. Further randomized controlled and large-scale trials would be required to confirm these findings. Finally, the personnel, protocol and facility in this study may reflect the local situation when a separate anesthesia team is available for sedative bronchoscopy.

## 5. Conclusions

The combination of dexmedetomidine in a sedation regimen met the safety and recovery demands of flexible bronchoscopy. Compared with the conventional midazolam-propofol-fentanyl regimen, the application of dexmedetomidine was associated with more stable sedation with less procedural pauses, shorter time to ambulation and higher bronchoscopist satisfaction.

## Figures and Tables

**Table 1:** The patient characteristics between both groups.

	Group D (*n* = 35)	Group M (*n* = 33)	*p* Value
Gender (M:F)	16:19	15:18	0.98
Age (year)	62.6 ± 12.9	63.6 ± 15.6	0.75
Weight (kg)	63.0 ± 13.2	57.4 ± 11.7	0.08
Height (cm)	161.9 ± 7.9	160.1 ± 8.7	0.38
ASA class I/II/III (*n*) ^1^	2000/12/23	2001/8/24	0.34
Cancer, *n*(%)	12 (34.2)	9 (27.3)	0.53
Baseline oxygenation (%)	96.8 ± 1.6	97.1 ± 2.8	0.61
Baseline heart rate (beats/min)	80.4 ± 11.5	80.3 ± 15.3	0.98
Baseline systolic blood pressure (mmHg)	148.9 ± 21.4	143.6 ± 24.1	0.34
Bronchoscopy examination/biopsy (*n*)	20/15	18/15	0.83
Sedation time (min)	29.2 ± 12.5	21.8 ± 10.3	0.02 *
Procedure time (min)	21.1 ± 11.0	18.4 ± 9.9	0.35

^1^ ASA: American Society of Anesthesiologists. * *p* values < 0.05. Age, weight, height and time were analyzed by t-test, other parameter were by Chi-square. The level of significance was set as *p* values < 0.05.

**Table 2:** The analysis of the sedation, procedure and safety profile between both groups.

	Group D (*n* = 35)	Group M (*n* = 33)	*p* Value
Propofol dose (mg)	147.4 ± 66.1	165.6 ± 88.3	0.37
Fentanyl dose (μg)	44.8 ± 14.8	46.9 ± 20.6	0.56
Dexmedetomidine (μg)	47.3 ± 16.1	0	NA
Midazolam dose (mg)	0	1.3 ± 0.8	NA
Procedural interruption by cough or movement, *n*(%)	1 (3.3)	12 (36.3)	<0.001 *
Bronchoscopist satisfaction ^1^ (*n*) 1/2/3/4	0/0/15/20	0/2/23/8	0.01 *
Transient hypoxemia, *n*(%)	8 (22.9)	6 (18.2)	0.63
Airway management			
Jaw lift, *n*(%)	7 (20)	8 (24.2)	0.67
Mask, *n*(%)	0 (0)	1 (3.0)	0.29
Intubation, *n*(%)	0 (0)	0 (0)	NA
Hypotension, *n*(%)	12 (33.3)	8 (24.2)	0.36
Bradycardia, *n*(%)	7 (20)	2 (6.1)	0.03 *

^1^ Bronchoscopist satisfaction 1/2/3/4 represents poor/fair/good/excellent. * *p* values < 0.05. Medication dose was analyzed by t-test and percentage parameters were by Chi-square. The level of significance was set as *p* values < 0.05.

**Table 3:** The anesthetic regimen and post-procedural outcome between both groups.

	Group D (*n* = 35)	Group M (*n* = 33)	*p* Value
Awake time (min)	6.4 ± 4.4	5.5 ± 4.5	0.14
Ambulation time (min)	24.9 ± 9.7	31.5 ± 11.9	0.016 *
Adverse events			
Nausea/vomiting, *n*(%)	4 (11.4)	1 (3.0)	0.18
Dizziness, *n*(%)	5 (14.2)	1 (3.0)	0.1
Recall, *n*(%)	0 (0)	0 (0)	NA
Pain, *n*(%)	1 (2.9)	0 (0)	0.33
Patient satisfaction ^1^ (*n*) 1/2/3/4	0/0/29/6	0/0/26/7	0.91

^1^ Patient satisfaction 1/2/3/4 represents poor/fair/good/excellent. * *p* values < 0.05. Time was analyzed by t-test, other percentages were by Chi-square. The level of significance was set as *p* values < 0.05.
